# Evaluating the effectiveness of the pre‑hospital trauma life support (PHTLS) program for the management of trauma patients in the pre-hospital emergency based on Kirkpatrick’s evaluation model

**DOI:** 10.1186/s12245-024-00589-2

**Published:** 2024-01-29

**Authors:** Mohammad Hadi Kamgar Amaleh, Sara Heydari, Peyman Nazari, Fatemeh Bakhshi

**Affiliations:** 1 International Campus, Shahid Sadoughi University of Medical Sciences, Yazd, Iran; 2 Emergency Nursing, Larestan University of Medical Sciences, Larestan, Iran; 3 Department of Medical Education, Medical Education and Development Center, Shahid Sadoughi University of Medical Sciences, Yazd, Iran; 4 Emergency Nursing, Gerash University of Medical Sciences, Gerash, Iran; 5 Nursing, Research Center for Nursing and Midwifery Care, Non-communicable Diseases Research Institute, Shahid Sadoughi University of Medical Sciences, Yazd, 8916877443 Iran

## Abstract

**Background:**

Pre-hospital trauma life support (PHTLS) training courses have been developed and widely adopted to enhance the proficiency of pre-hospital personnel in handling trauma patients. The objective of this study was to assess the effectiveness of the educational program for managing trauma patients in the pre-hospital emergency setting, utilizing Kirkpatrick’s educational evaluation model.

**Methods:**

This is an observational approach, consisting of four sub-studies. The PHTLS course was conducted over a 2-day period, encompassing both theoretical and practical components. For this study, we selected pre-hospital personnel from three emergency aid stations using a convenient sampling method. These personnel underwent their first-ever PHTLS course training, and we subsequently analyzed the effectiveness of the training program using Kirkpatrick’s four levels of evaluation: satisfaction, learning, behavior, and results.

**Results:**

The study conducted on Kirkpatrick’s first-level analysis revealed that participants expressed a high level of satisfaction with the quality of all aspects of the course. Moving on to the second and third levels, namely learning and behavior, significant improvements were observed in the average scores of various skills that were examined both immediately after the course and 2 months later (*P* < 0.05). However, when it comes to the fourth level and the impact of the course on indicators such as mortality rate and permanent disability, no significant changes were observed even after an average of 3 months since the course was introduced.

**Conclusion:**

The implementation of PHTLS has been linked to the enhancement of participants’ skills in treating trauma patients, leading to the application of acquired knowledge in real-life scenarios and a positive change in participants’ behavior. The evaluation of PHTLS courses in Iran, as in other countries, highlights the need for specialized training in pre-hospital trauma care. To ensure the continued effectiveness of the PHTLS course, it is advisable for managers and policymakers to encourage regular participation of PHTLS employees in the program.

**Supplementary Information:**

The online version contains supplementary material available at 10.1186/s12245-024-00589-2.

## Key messages

What is already known on this subject

• Pre-hospital trauma life support (PHTLS) serves as a standardized algorithm for managing trauma patients.

• The PHTLS course successfully enhances prehospital personnel trauma management skills leading to improved learning and behavior change.

What this study adds

• Repeating the course annually may enhance its effectiveness, ensure stability in behavioral changes, and reduce the impact on the mortality rate of trauma patients.

• Various aspects of the PHTLS course, based on Kirkpatrick’s evaluation model, have been confirmed to have a significant impact on both learning outcomes and behavioral change.

**Supplementary Information:**

The online version contains supplementary material available at 10.1186/s12245-024-00589-2.

## Introduction

Trauma-related complications are most prevalent (ranging from 50 to 70%) immediately following an injury, at the accident scene, and during the initial hours of hospitalization [1, 2]. It is widely recognized that the initial treatment administered in trauma cases has a profound impact on mortality rates [3]. The effectiveness of prehospital care hinges on the competence and expertise of emergency medical services (EMS) personnel [4]. Pre-hospital trauma life support (PHTLS) serves as a standardized algorithm for managing trauma patients. This algorithm, presented as a comprehensive training course, aims to minimize patient injuries and fatalities by delivering exceptional care [5].

The PHTLS course aims to establish a high level of compatibility between prehospital and inhospital care standards [6]. Over the years, this course has gained widespread global implementation and been adopted in numerous countries [7, 8]. In recent years, with the backing of the Emergency Organization of Iran and a design closely aligned with the international version, this course has been conducted through 2-day theoretical and practical workshops [9].

The implementation of PHTLS has shown promising potential effects across the globe [10]. Esmaeilzadeh et al. (2022) propose that the incorporation of PHTLS training programs into prehospital emergency medical service systems is an unavoidable step [8]. However, there is a lack of comprehensive data on the long-term impact of this training course on disability and mortality rates within communities. It is crucial to evaluate the effectiveness of PHTLS based on educational indicators rather than solely relying on short-term outcomes like knowledge level or on-scene management [11]. By utilizing the Kirkpatrick model to assess the effectiveness of continuing education programs, informed decisions can be made regarding program continuation and identifying areas for improvement [12].

In order to ensure accurate program evaluation, it is essential to utilize a valid evaluation model. One widely recognized model is Kirkpatrick’s evaluation model (KM), which is structured in four stages [13]: satisfaction, learning, behavior, and results. Level 1 (satisfaction): This stage focuses on gathering the opinions of course learners regarding the course. Level 2 (learning): This stage assesses whether the course has effectively facilitated learning and increased the knowledge of the learners. Level 3 (behavior): This stage examines whether the course has influenced a change in the behavior of the learners. Level 4 (results): This stage evaluates the impact of conducting this course on organizational indicators [14, 15]. By employing Kirkpatrick’s evaluation model, program evaluators can ensure a comprehensive and reliable assessment of the program’s effectiveness.

In summary, the response level measures how much participants find the training appealing and captivating. The level of learning evaluates the knowledge and skills acquired by participants during the training. The behavior level involves superiors and peers assessing whether trainees are applying what they have learned. The outcome level determines if participants have achieved the learning outcome or goal [16]. To address the research questions and utilize the chosen model, the present research was conducted through multiple sub-studies. At each stage of the model, either descriptive and observational studies were employed based on the specific objectives. Overall, this research aims to comprehensively assess the participants’ engagement, learning, application, and achievement in relation to the training program. By utilizing a combination of descriptive and analytical studies, a thorough understanding of the effectiveness and impact of the training can be obtained.

In light of the existing body of knowledge, it is surprising that no previous study has evaluated the effectiveness of this course using an educational evaluation model. Consequently, we have undertaken a groundbreaking study to ascertain the true impact of the trauma patient management training program in the prehospital emergency aid bases of Lar city, Fars Province, Iran, from 2022 to 2023. By employing Kirkpatrick’s renowned educational evaluation model, we aimed to provide a comprehensive and rigorous assessment of the program’s efficacy.

## Methods

### Study design

This study is an observational research conducted from 2022 to 2023 in collaboration between Shahid Sadoughi University of Medical Sciences, Yazd, Iran, and the emergency aid bases of Lar city. The research was divided into four sub-studies, each based on the Kirkpatrick evaluation model and research questions. Each step of the model utilized a different type of observational design to achieve the objectives. This study was conducted in accordance with the Declaration of Helsinki and after obtaining ethical approval from the Ethics Committee of Shahid Sadoughi University of Medical Sciences and Health Services of Yazd (IR.SSU.REC.1401.099). All participants willingly completed the informed consent form.

### Study participants and sampling

This study focused on EMS workers stationed at three prominent emergency aid bases in Lar city, Fars province. To be included in the research, participants were required to have no prior training in the PHTLS method and hold a university degree in emergency medicine or nursing. Given the limited number of individuals (55 individuals) across the three bases, the entire community was involved in the study, eliminating the need for sampling. Therefore, in calculating the sample size, we had no choice but to consult Krejcie and Morgan’s table [17]. As a result, a sample size of 48 was determined.

### PHTLS educational course

A trauma training course, based on the pre-hospital trauma life support (PHTLS) program, was conducted at the Lar Emergency Training Center over 2 days. The course covered various topics including scene assessment, primary assessment, airway management, breathing and ventilation, bleeding and shock, brain injury, spinal cord injury, secondary assessment, trauma management in children, trauma management in the elderly, trauma management in pregnant women, burns, triage, and resuscitation.

The primary objective of this workshop-style course was to enhance the skills of pre-hospital emergency personnel. Each course had a maximum capacity of 25 participants, who were selected from different bases in Lar city. Two workshops were conducted as part of this plan, with the participants attending for the first time. The instructors for these workshops were carefully chosen from the fields of emergency medicine, health in disasters and crises, nursing, and emergency medicine.

### Kirkpatrick evaluation model

The Kirkpatrick model was utilized to assess the program. This model serves as the theoretical framework for the current research. It was specifically designed to evaluate educational courses, with a primary focus on learning outcomes and effectiveness [18]. The participants of the first to third levels were the same. Further information regarding the application of each level of the model is outlined below:

#### Level 1: Satisfaction

The methodology employed in this stage was descriptive and cross-sectional, aiming to comprehensively understand the participants’ satisfaction. To accurately measure their satisfaction, a researcher-designed tool was crafted. This involved an extensive review of relevant literature and library studies, focusing on previous research that explored satisfaction levels in training courses. To ensure the tool’s psychometry, a two-pronged approach was adopted. Firstly, face validity was assessed through individual interviews, where participants shared their valuable insights on the difficulty level, appropriateness, and clarity of the items. Secondly, the relevance, clarity, and simplicity of the satisfaction questionnaire were assessed using the content validity index (CVI). The CVI scores for relevance, clarity, and simplicity were found to be 0.95, 0.85, and 0.95, respectively.

#### Level 2: Learning

The purpose of this stage was to observe and compare the participants’ performance before and after the training course. The level of learning was assessed by conducting the objective structured clinical examination (OSCE) test in nine stations. To perform the OSCE test, we used nine clinical skill scenarios regarding trauma patients which were developed and validated in Najafi et al.’s study [19]. For data collection and assessment in each station, we utilized a standard trauma clinical assessment checklist which was adapted from Shakeri et al.’s study [20]. This checklist included nine skills focused on the participants’ ability to handle traumatized individuals:

1. Examining a trauma patient (43 points)
2. Restricting spinal movement in a seated patient (12 points)
3. Restricting spinal movement in a patient lying down (14 points)
4. Restricting movement of an injured long bone (10 points)
5. Restricting movement of an injured joint (9 points)
6. Utilizing a stretching splint (14 points)
7. Controlling bleeding and treating shock (7 points)
8. Inserting oral-pharyngeal and nasopharyngeal airways and performing suctioning (13 points)
9. Performing oral-tracheal ventilation and intubation (27 points)

Each skill was evaluated separately, and the total score ranged from 0 to 149, with a higher score indicating greater proficiency. The checklists’ face and content validity were assessed and confirmed by a panel of 10 experts. The tool’s reliability was assessed using the Kuder-Richardson 20 criterion, which yielded a coefficient exceeding 0.8. This indicates a high level of reliability for the tool [19, 20].

During the OSCE test, participants were provided with scenarios at each station. Roughly 7 min were dedicated to performing each skill at every station. The research team evaluated participants performance using the trauma clinical assessment checklist with evaluators who were not part of the team.

#### Level 3: Behavior

The objective of this stage was to closely observe and compare the behavioral patterns of employees, in comparison to the previous level. The primary focus was to evaluate their clinical skills by closely monitoring their performance in a real-life environment. In order to attain this, the trauma clinical assessment checklist was once again employed. The performance of the participating personnel was meticulously observed for a duration of 1 to 3 months (2 months on average) following the completion of the course.

To ensure the utmost accuracy and consistency in the evaluation process, this stage was designed as a DOPS (direct observation of procedural skills) test. Before implementing the third step, three evaluators, who were slated to be present at the base and accompany the trauma mission, underwent a comprehensive simulated workshop evaluation conducted by an esteemed emergency medicine specialist. The inter-rater agreement was assessed which was above 80% and was then confirmed.

#### Level 4: Results

To assess the effectiveness and outcomes of the course, a descriptive study was conducted. The process of data collection at this particular level differed from the previous three levels of study participants. Instead, we gathered information specifically about trauma victims who were attended to by the participants at the scene of the incident. A researcher-made survey was utilized to gather information on organizational indicators. By conducting a literature review and extracting pertinent themes, we ensured that our survey encompassed a comprehensive range of factors. This approach allowed us to gather valuable data on patients’ demographics, medical condition, and treatment outcomes (especially the recovery rate after 2 weeks and the occurrence of permanent disabilities following trauma). To maintain content validity, a panel of experts—10 faculty members (5 from nursing and five from emergency medicine)—assessed the surveys. Face and content validity was assessed. Clarity and wording were refined. The overall CVI score was more than 0.85 [21]. It is important to note that this data was completely anonymous, with no patient names or identifiable characteristics included. The collected information focused on treatment details and the clinical progress of each individual. Following the methodology outlined in Mangat et al.’s study (2021), the indicators were assessed, and calculations were performed for each patient over 2 weeks [22]. The information for all trauma patients was collected within 3 months after the course, and this data was then compared in 2- to 5-month period (3 months on average) after the course.

### Statistical analysis

The Kolmogorov–Smirnov test serves as a valuable tool in examining the normal distribution of variables. To analyze the data, descriptive statistical methods were employed, while the paired *t*-test was utilized for comparing variables within the same group. The data analysis was conducted using the SPSS V.21 software, renowned for its comprehensive capabilities. A significance level of less than 0.05 is considered significant.

## Result

In this study, a total of 48 EMS personnel participated in the evaluation of the PHTLS course, utilizing levels 1 to 3 of Kirkpatrick’s evaluation model. The participants’ average age was found to be 35.29 ± 6.52 years.

### Results level 1: Satisfaction

Table 1 displays the results obtained from the satisfaction questionnaire for the PHTLS course, using Likert scale items. The items were classified into five dimensions: implementation of the practical part of the course, food and hospitality during the course, schedule of course implementation, course location, and course executive staff and instructors. Each dimension’s questions aimed to assess participants’ satisfaction with the quality of that specific aspect. The questionnaire’s items were scored on a scale of 1 to 4.

**Table 1:** Mean and standard deviation of participants’ responses to self-administered Likert scale questions, categorized by each dimension of the PHTLS course

Workshop dimension		Mean (SD)^a^
**Implementation of the practical part**		3.45 ± 0.43
**Food and hospitality during the course**		3.71 ± 0.5
**Schedule of course implementation**		3.56 ± 0.35
**Course location**		3.57 ± 0.43
**Course executive staff**		3.65 ± 0.48
**Instructors**	First	3.67 ± 0.6
	Second	3.20 ± 0.69
	Third	3.35 ± 0.67
	Forth	3.48 ± 0.66
	Fifth	3.02 ± 0.75

^a^Range: 1–4

### Results levels 2 and 3: Learning and behavior

This section presents the findings regarding the learning and behavior of participants in the PHTLS course. The aim was to assess the participants’ progress before and after the course (learning level) and 2 months after the course (behavior level). The scores reported here are based on the observation checklist used in Kirkpatrick’s second- and third-level assessments. Each skill was evaluated at least five times per person using a checklist and the DOPS method.

Table 2 compares the learning scores of PHTLS course participants before and after the course (learning level) and the average 2 months after the course (behavior level). The comparison of average scores before and after the training in the learning level for all skills showed significant improvement (*P* < 0.05). Furthermore, the comparison of average scores before the training (learning level) and 2 months after the course (behavior level) also revealed significant changes in all skills (*P* < 0.05). However, when comparing the average scores after the training (learning level) with the average 2 months after the course (behavior level), no significant changes were observed (*P* > 0.05).

**Table 2:** Comparing the learning scores of PHTLS course participants before and after the course (learning level) and the average 2 months after the course (behavior level)

Skill	Comparison before and after training (learning level)			Comparison of pre-training (learning level) and behavior level			Comparison of after-training (learning level) and behavior level		
	Before course	After course	*p*-value	Before course	Average 2 months	*p*-value	After course	Average 2 months	*p*-value
**The skill of examining/managing trauma patients**	18.5 ± 3.32	62.33 ± 6.57	0.00	18.5 ± 3.32	34.29 ± 5.8	0.00	62.33 ± 6.57	34.29 ± 5.8	0.228
**The skill of airway-oral-pharyngeal, nasal-pharyngeal, and suctioning**	5.81 ± 1.19	10.65 ± 1.96	0.00	5.81 ± 1.19	10.8 ± 1.96	0.00	10.65 ± 1.96	10.8 ± 1.96	0.37
**Bleeding control/shock treatment skills**	3.58 ± 0.76	5.93 ± 0.98	0.00	3.58 ± 0.76	5.86 ± 0.9	0.00	5.93 ± 0.98	5.86 ± 0.9	0.271
**The skill of using a Traction splint**	5.68 ± 1.2	10.8 ± 1.83	0.00	5.68 ± 1.2	11.21 ± 2.06	0.00	10.8 ± 1.83	11.21 ± 2.06	0.78
**The skill of limiting the patient in the supine position**	5.69 ± 1.22	10.24 ± 1.6	0.00	5.69 ± 1.22	10.82 ± 2.4	0.00	10.24 ± 1.6	10.82 ± 2.4	0.732
**The ability to limit the movement of the spine of a sitting patient**	5.17 ± 1.23	8.7 ± 1.22	0.00	5.17 ± 1.23	9.43 ± 1.77	0.001	8.7 ± 1.22	9.43 ± 1.77	0.94
**The skill of limiting the movements of the injured long bone**	4.36 ± 0.73	7.8 ± 1.22	0.00	4.36 ± 0.73	8.02 ± 1.15	0.00	7.8 ± 1.22	8.02 ± 1.15	0.299
**The ability to limit the movement of the injured joint**	3.93 ± 0.63	8.38 ± 2.38	0.00	3.93 ± 0.63	7.02 ± 1.31	0.00	8.38 ± 2.38	7.02 ± 1.31	0.812
**Endotracheal ventilation/intubation skills**	12.19 ± 1.62	19.45 ± 2.66	0.00	12.19 ± 1.62	21.51 ± 3.58	0.003	19.45 ± 2.66	21.51 ± 3.58	0.695

### Results level 4: Evaluating course effectiveness

At this level of Kirkpatrick’s evaluation, we delve into a wider dimension to assess the effectiveness of the desired training. However, it is important to note that this level is challenging due to the need to consider various components related to organizational performance [23]. In our study, we focused on two key aspects: the rate of accidents and disabilities up to 2 weeks after the incident, as well as the management provided by the EMS team. To analyze these factors, we compared the frequency of missions, mortality rates, and permanent disabilities 3 months before and 3 months after the training course. Table 3 shows frequencies of these factors for trauma patients. The goal was to determine changes in these variables.

**Table 3:** Frequencies of total missions, trauma missions, mortality, and incidence of permanent disability of trauma patients 3 months before and 3 months after the course

Variable	Total missions	Trauma mission	Mortality of trauma victims	Permanent disability
Three months before PHTLS	612	271 (44.28)	23 (8.48)	17 (6.27)
Three months after PHTLS	600	260 (43.33)	20 (7.69)	15 (5.76)

## Discussion

Prehospital care plays a crucial role in enhancing the outcomes of trauma patients. As a result, EMS personnel must possess exceptional skills to provide optimal on-scene care during the critical golden time. To enhance the knowledge and technical proficiency of employees, PHTLS courses are being implemented in various regions worldwide [8]. This study aims to examine the effectiveness of this comprehensive course on staff skills and the outcomes of trauma patients, utilizing the Kirkpatrick evaluation model.

The utilization of Kirkpatrick’s evaluation model is a significant advantage of this study. To the best of our knowledge, this study represents the initial instance in which all four levels of this model have been employed to evaluate the efficacy of the PHTLS training algorithm and course. Such comprehensive evaluations of training courses, encompassing a high level of learning, serve to assist policymakers and educators in identifying areas of improvement and enhancing these training programs [14].

To examine the initial level of the model, we assessed the satisfaction of prehospital personnel who took part in the course. Each dimension of the questionnaire was scored on a scale from 1 to 4, and the average total scores for each dimension exceeded 3. This outcome suggests that the participants were satisfied with the course. Notably, the employees expressed high satisfaction with the quality of the PHTLS practical course and the teaching instructors.

The comparison of scores in the assessed skills revealed significant improvements in the participants’ learning at the second level. When comparing scores before the course (second level or learning) to those taken on average 2 months after (third level or behavior), significant changes were observed. This suggests that the participants’ behavior had been positively influenced by their learning experience. In essence, the results confirmed that the changes made in the participants’ behavior were lasting and impactful.

Overall, the model effectively evaluated the participants’ learning and its application in real-life situations. The results demonstrated significant improvements in both learning and behavior, highlighting the effectiveness of the course in bringing about meaningful changes. According to Frank et al., the PHTLS course has been shown to enhance the knowledge and confidence of employees [24]. A recent study revealed that emergency medical services student skills are positively affected by the PHTLS course [7]. Falaki and colleagues also confirmed that the introduction of PHTLS led to an improvement in knowledge and skills [25]. However, the long-term effects of this training have been a subject of debate among Haske et al., with some suggesting that the improvements are only temporary [6]. In this study, we also assessed the consolidation of learning for behavior but only for an average period of 2 months. It appears that PHTLS should be repeated through continuous training programs, and EMS personnel should participate in it annually [25].

To assess the effectiveness of the PHTLS course at level 4, mortality and permanent disability were examined. Although statistical analysis was not employed, the resulting frequencies showed minimal variation, and the changes were not easily noticeable. Similarly, Blomberg et al. also found no significant impact on the mortality of injured individuals after implementing the PHTLS course [26]. However, Teuban et al., in a comprehensive 7-year study, reported an improvement in the efficiency of prehospital care following the introduction of PHTLS in European cities [5]. Additionally, the study conducted by Ali et al. demonstrated a decrease in the mortality rate of multi-trauma patients after implementing PHTLS courses [27]. The disparity in these results may be attributed to the different organizational indicators examined and the varying duration of follow-up to assess the effects of the PHTLS course. However, none of the studies provided specific findings regarding the impact of PHTLS on other trauma complications, such as permanent disabilities.

### Strengths and limitations

The present study, like many others, had its limitations. It was conducted as a student thesis for a master’s degree in emergency nursing, which means that the period for data collection was limited. Consequently, we were unable to thoroughly examine the third and fourth levels of Kirkpatrick’s model over an extended period. Furthermore, the scope of this study was confined to a specific geographical area in Iran, which unfortunately experiences a high number of road accidents. Additionally, despite our efforts to include as many emergency medical services (EMS) employees as possible, we were only able to sample from three major emergency aid bases in that particular area. As a result, our sampling process was limited to convenience sampling, and we were unable to gather data from a wider range of employees or across multiple geographical areas.

It is important to acknowledge these limitations as they may impact the generalizability of our findings. However, despite these constraints, we believe that our study still provides valuable insights into the field of emergency and contributes to the existing body of knowledge.

## Conclusion

The present study has revealed that participants were highly satisfied with the quality of the implementation of the PHTLS course under current conventional conditions. Furthermore, it has been confirmed that the PHTLS course successfully enhances various skills taught in PHTLS, leading to improved learning and behavior change. This study highlights the effectiveness of utilizing Kirkpatrick’s model for analyzing and evaluating educational courses.

However, to ensure the continued effectiveness of the PHTLS course, it is advisable for managers and policymakers to encourage PHTLS employees to regularly participate in the course. Additionally, it is recommended to conduct further studies to evaluate the effectiveness of this course, as well as similar courses, in managing trauma patients. These studies should consider a broader range of indicators related to the prognosis of trauma patient management, rather than solely focusing on mortality rates. Overall, this research underscores the importance of continuous professional development and the need for ongoing evaluation to enhance the quality of trauma patient care. By implementing these recommendations, healthcare organizations can further improve the outcomes and overall management of trauma patients.

### Supplementary Information

**Additional file 1.** STROBE Statement—Checklist of items that should be included in reports of cross-sectional studies.

## Data Availability

The datasets generated during and/or analyzed during the current study are available from the corresponding author on reasonable request.
